# High burden of hypovitaminosis D among the children and adolescents in South Asia: a systematic review and meta-analysis

**DOI:** 10.1186/s41043-022-00287-w

**Published:** 2022-03-17

**Authors:** Mahbubul H. Siddiqee, Badhan Bhattacharjee, Umme Ruman Siddiqi, Mohammad Meshbahur Rahman

**Affiliations:** 1 Department of Mathematics and Natural Sciences, School of Data and Sciences, BRAC University, Dhaka, 1212 Bangladesh; 2 Research Wing, Red & White Innovations, Mirpur DOHS, Dhaka, 1216 Bangladesh; 3 Communicable Disease Control Unit (CDC), Directorate General of Health Services, Dhaka, 1212 Bangladesh; 4 Biomedical Research Foundation, Dhaka, 1230 Bangladesh

**Keywords:** Vitamin D, Deficiency, Insufficiency, Hypovitaminosis, Prevalence, Children, Adolescents, South Asia, Systematic review

## Abstract

**Background:**

Vitamin D is vital for the growth and development of children. While deficiency and/or insufficiency of vitamin D among South Asian children are frequently reported in the literature, the lack of a meta-analysis has left its true extent poorly characterized. In this study, we aimed to conduct a systematic review and perform meta-analyses of the prevalence of hypovitaminosis D among the children of the South Asian countries.

**Methods:**

Two major electronic search engines (PubMed and Scopus) and one database (Google scholar) were used; original studies, conducted among South Asian children and adolescents and published between 1 January 2001 and 31 December 2019. A random-effect meta-analysis was also performed to calculate the pooled prevalence of hypovitaminosis D followed by subgroup analyses for countries and age groups.

**Results:**

After applying inclusion and exclusion criteria, a total of 41 studies with a total population size of 18,233 were finally selected. The overall prevalence of hypovitaminosis D was 61% [95% CI: 46% to 71%] with highly significant heterogeneity (*I*^2^ = 99.72%; *p* < 0.0001). The average level of serum vitamin D ranged from 5 ng/mL to 34 ng/mL, with a weighted mean of 19.15 ng/mL (weighted standard deviation 11.59 ng/mL). Country-wise analysis showed that hypovitaminosis D in Afghanistan was the highest [96.2%; 95% CI: 91% to 99%], followed by Pakistan [94%; 95% CI: 90% to 96%], India [64%; 95% CI: 46% to 79%], Bangladesh [35.48%; 95% CI: 32% to 39%], Nepal [35%; 95% CI: 1% to 83%], and Sri Lanka [25%; 95% CI: 16% to 36%]. Age group analyses revealed that hypovitaminosis D was most prevalent among neonates [85%; 95% CI: 76% to 91%], followed by school-going children [57%; 95% CI: 33% to 80%], and preschool children [55%; 95% CI: 35% to 75%].

**Conclusion:**

This study generates quantitative evidence and specific extent of hypovitaminosis D in the South Asian countries as a public health concern. Being the first systematic review for this region, results from this study will create awareness and will facilitate adopting mitigation strategies by the policymakers and the governments to address this problem.

**Supplementary Information:**

The online version contains supplementary material available at 10.1186/s41043-022-00287-w.

## Background

Vitamin D has a widespread role in the early development of children. The deficiency of this vitamin can often lead to suboptimal bone mass in infants, children, and adolescents. Nutritional rickets is a major bone-related disease that is caused by vitamin D deficiency [1–3]. According to the World Health Organisation (WHO), the peak incidence of rickets occurs among children and adolescents aged 2–11 years [4]. Besides rickets, vitamin D deficiency can also cause osteomalacia and other bone-related deformities among children [3, 4]. It has been suggested that up to 200 genes could be regulated by the active form of vitamin D (1,25 dihydroxy vitamin D), indicating its highly pleiotropic role [2, 5]. Moreover, study reports suggested that the biological plausibility of vitamin D deficiency is also correlated with various kinds of chronic diseases like diabetes, cardiovascular disease, cancer, tuberculosis, etc. [2, 5–7] So, a child suffering from vitamin D deficiency early in life becomes more susceptible to other kinds of diseases in the latter part of life.

Vitamin D deficiency affects as many as 1 billion people globally and 50% of the world population suffers from vitamin D insufficiency [2, 5]. Worldwide, the extent of vitamin D deficiency among children and adolescents in both developed and developing countries is highly variable; prevalence as low as 5% to as high as 95% of the study population has been reported [8–10].

A variety of factors have been highlighted as underlying variables to explain this large variance in serum vitamin D status; besides nutrition, the extent of sunlight exposure is arguably the most important determinant [2, 11]. Sunlight exposure eventually depends on some other factors like geographical location, people's skin colour, attitude towards sunlight exposure, clothing practice, etc. [2, 5, 12]. As such, understanding comparative variation within a region may reveal crucial clues regarding the potential determining factors of vitamin D deficiency or insufficiency (hypovitaminosis D).

South Asia consists of eight countries—India, Bangladesh, Pakistan, Nepal, Bhutan, Maldives, Sri Lanka, and Afghanistan. Together, these South Asian countries occupy an area of 5.131 million square kilometres and have a population of about 1.8 billion [13, 14].

According to UNICEF, around 627 million children (< 18 years of age) live in South Asian countries and cover up approximately 36% of the total population (1.8 billion) [15]. Reported data indicates a high prevalence of nutritional rickets and other bone-related diseases, cardiovascular problems, diabetes, acute respiratory infections, tuberculosis, and other communicable diseases among South Asian children [4, 16–18]. All of these can be potentially linked to the high prevalence of hypovitaminosis D in this region.

Despite the possibility of large-scale hypovitaminosis D in South Asia, we found only one systematic review (on Indian adolescent girls based on a very limited number of studies) in this region [19]. To address this knowledge gap, this study aims to conduct a systematic review and meta-analysis on the prevalence of hypovitaminosis D among South Asian children and adolescents.

## Methods

Preferred Reporting Items for Systematic Reviews and Meta-Analyses (PRISMA-P 2015) have been followed as recommendations and guidelines for conducting this systematic review [20]. Since, we did not register the review protocol anywhere (PROSPERO, Cochrane, etc.), a completed copy of the PRISMA checklist (PRISMA 2020) has been added as an Additional file 2.

### Data source and search strategy

PubMed and Scopus were the main databases, and Google Scholar was the main search engine, used in this analysis (to prevent personalized results, the search was conducted, after logging out of all Google accounts). Two researchers (BB and MMR) independently investigated these three datasets (from 26 October 2019 until 26 January 2020) to find out the studies conducted from 2001 until the search date. The details about search strategy, original MeSH terms, and alternative terms are available in Additional file 1: Table S1.

The searches were carried out in English. The corresponding author's personal profiles available online (Google Scholar, ResearchGate, ORCID, and organizational profile), as well as the reference list of our selected studies, were further explored to maximize the search efficiency.

To ensure the inclusion of grey literature, we went through online archives of newspapers that have been published in English among South Asian countries as; The Hindu, The Indian Express, New Age, The Nation, Daily Bhutan, Maldives Times, Himalayan Times, Sunday Observer, etc. We also explored governments’ reports and published abstracts (in electronic media) as relevant sources from the conference held in South Asian countries.

### Study selection criteria

Clinical Practice Guideline of the Endocrine Society currently defines vitamin D deficiency, insufficiency, and sufficiency as a serum level of vitamin D < 12 ng/mL (< 30 nmol/L), < 20 ng/mL (< 50 nmol/L) and > 20 ng/mL (> 50 nmol/L), respectively [21, 22]. In this study, we defined hypovitaminosis D (vitamin D deficiency or insufficiency) as per current guidelines (cut-off < 20 ng/mL). Studies were selected if serum levels of vitamin D < 20 ng/mL in South Asian children and adolescents were reported. The inclusion criteria were: study conducted in South Asian countries from 1 January 2001 to 31 December 2019; study conducted in the hospital setting or community setting among apparently healthy children and adolescents who were up to 18 years of age, children and adolescents with a minor illness whose physical conditions were not correlated with any chronic diseases or coexisting morbidity [e.g. chronic kidney disease, cardiovascular disease, diabetes, rheumatoid arthritis, cancer, tuberculosis, body aches and pain, proximal muscle weakness, osteoporosis, etc.]. Among different study designs, cross-sectional, longitudinal, case–control (only control group), randomized clinical trial (baseline and placebo data) were included.

Studies were excluded if they had a sample size less than 50 [19]; reported vitamin D levels after some form of intervention or supplementation; conducted on other groups of the population rather than children and adolescents such as pregnant women, adults, and elderly; the reported prevalence of hypovitaminosis D associated to any kind of chronic diseases or disease related to any coexisting morbidity; conducted on a special group of children and adolescents such as physically or mentally challenged; did not mention the prevalence of deficiency and mean level of serum vitamin D; letter to the editor, review article, editorial article; Studies which satisfied selection criteria but were not obtainable from the authors after request were also excluded.

To handle the references and prevent duplications, Mendeley Desktop Program (version 1.19.4) was used. After eliminating duplicates, two researchers (MHS and URH) independently reviewed all papers before final selection for meta-analysis. Any disagreements were resolved through discussion with co-authors.

### Data extraction

To extract data from all eligible studies, a standardized form was used. The following information has been collected for each study: publication details [e.g. first author, publication date, journal name, and publisher]; research setting, design and population [e.g. country, study area, study design, method of measurements, and sample size]; participants’ characteristics [e.g. gender, age, and socio-economic status] and major findings [mean level of serum vitamin D and prevalence percentage of hypovitaminosis D]. Data extraction was independently performed by two researchers (BB and MMR) and subsequently, it was cross-checked by two other researchers (MHS and URS). In our selected studies, where mean values of serum vitamin D were given in nanomol per unit litre (nmol/L), we converted to nanogram per millilitre (ng /mL) by dividing with 2.5 (according to the international unit conversion system) to maintain uniformity of data.

We resolved any disputes between us through discussion during data selection and data extraction. Eventually, all four researchers fully agreed to the selected studies before the data extraction began. Therefore, no statistical analysis of inter-rater agreements was performed.

### Evaluation of study quality

We used a checklist of 10 parameters validated by Hoy et al. (2012). To assess the risk of bias (as weak, moderate, and high) for the selected papers [23]. As per the checklist, studies with a score of 0–3 are considered as low risk, a score within 4–6 is moderate risk, and studies with a score of 7–9 are considered as high risk of bias.

### Statistical analysis

The mean value of the serum vitamin D and the prevalence of hypovitaminosis D in South Asian children and adolescents are regarded as summary measurements. The weighted mean level of serum vitamin D was calculated by using Microsoft Excel (version 2016). A random-effect meta-analysis was used to obtain the weighted pooled prevalence with a confidence interval of 95%. Cochran’s Q test and the *I*^2^ statistics were used to assess heterogeneity [24]. Substantial heterogeneity was suggested with an *I*^2^ of more than 75% [25]. The analyses were performed using the metaprop, metabias, metafunnel commands by Stata version 15 (Stata Corp, College Station, TX).

## Results

**Fig. 1:**
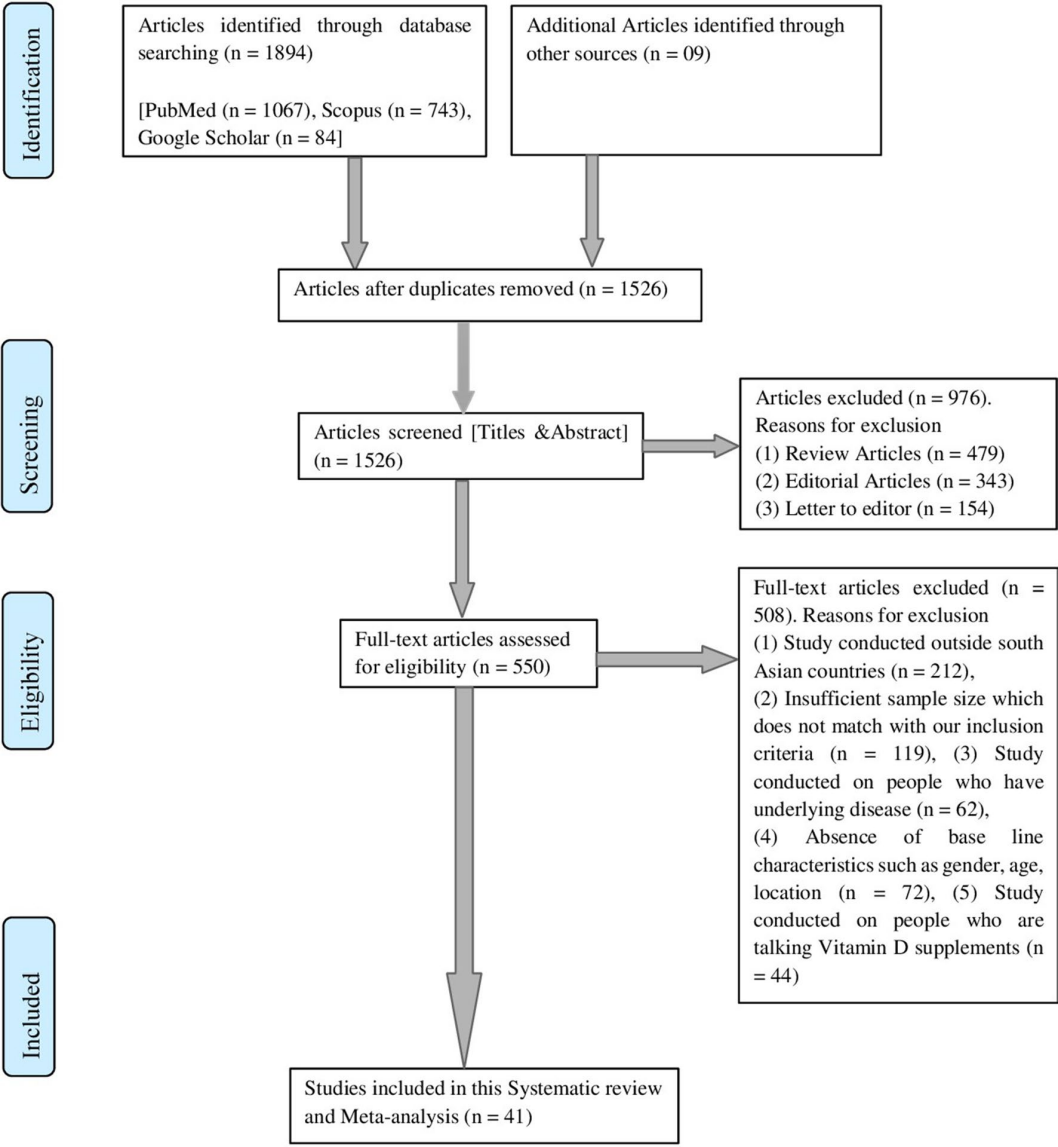
PRISMA chart showing the flow of information through the different phases of the systematic review

A total of 1903 articles were retrieved from different databases by using our search strategy. Among these 1903 studies, 1862 articles were excluded because they did not fulfil our inclusion criteria. A total of 41 articles, finally qualified for meta-analyses. Figure 1 shows the selection process used in this study.

The study design was cross-sectional for most of the studies (30 out of 41) and the rest were either case–control or randomized control trials. Furthermore, more than half of the selected studies did not mention demographic area (25 out of 41), and socio-economic condition (26 out of 41) for the study population. Table 1 shows the summary outlining the characteristics of selected articles.

**Table 1:** Characteristics of selected study articles

Authors	Year	Country	Study area (urban or rural)	Study design	Age	Gender	Socio economic status	Vitamin D estimation method	Sample size (N)	Average level of vitamin D (ng/mL)	Standard deviation (S.D.)
Marwaha et al. [26]	2005	India	Urban	Community—CS	10–18 y	Both	Both	RIA	5137	11.8	7.2
Sahu et al. [27]	2008	India	Rural	Community—CS	10–20 y	Both	Lower	RIA	155	20.16	9
Mandlik et al. [28]	2018	India	Rural	Community—CS	6–12 y	Both	NM	ELISA	359	23.4	4.12
Kapil et al. [29]	2017	India	NM	Community—CS	6–18 y	Both	Both	CLIA	626	11.8	5.475
Basu et al. [30] *	2014	India	Both	Hospital—CS	6–16 y	Both	NM	CLIA	154	13.5	12.22
Puri et al. [31]	2007	India	NM	Community—CS	6–18 y	Girls	Both	RIA	404	12.748	6.172
Kadam et al. [32]	2011	India	NM	Community—CS	8–12 y	Girls	Lower	RIA	214	24.74	10.48
Khadgawat et al. [33]	2013	India	NM	Community—RCT	10–14 y	Both	NM	CLIA	713	11.69	5.36
Chaudhuri et al. [34]	2016	India	NM	Hospital—CC	8–18 y	Both	NM	CMIA	50	22.5	NM
Sharawat et al. [35]	2019	India	Rural	Community—CS	5–10 y	Both	Lower	IRMA	100	17.652	9.912
Marwaha et al. [36]	2015	India	NM	Community—CS	10–15 y	Both	NM	CLIA	205	6.3	4.6
Sarma et al. [37]	2019	India	Both	Community—CS	8–14 y	Both	NM	RIA	500	37.7	0.93
Sanwalka et al. [38]	2012	India	NM	Community—CS	15–18 y	Girls	NM	RIA	120	8.88	4.384
Mandlik et al. [39]	2017	India	Rural	Community—RCT	6–12 y	Both	NM	ELISA	106	23.84	4.44
Borker et al. [40]	2009	India	NM	Hospital—CC	6–12 y	Both	NM	HPLC	50	26.16	12.28
Garg et al. [41]	2013	India	Urban	Community—CS	Mean age 14 y	Both	NM	RIA	1829	8.3	5.2
Khadilkar et al. [42]	2012	India	NM	Community—CS	8–12 y	Girls	Lower	RIA	214	24.73	10.65
Patel et al. [43]	2015	India	NM	Community—CS	10–14 y	Both	Both	CMIA	181	17.7	6.7
Marwaha et al. [44]	2017	India	NM	Community—CS	6–18 y	Girls	NM	CLIA	847	9.9	5.6
Prasad et al. [45]	2016	India	NM	Hospital—CC	< 3 y	Both	NM	ELISA	61	31	8.7
Agarwal et al. [46]	2012	India	NM	Hospital—CS	NM	Both	NM	RIA	336	6.15	NM
Basu et al. [30] *	2014	India	Both	Hospital—CS	1–5y	Both	Lower	CLIA	156	23	16.29
Wayse et al. [47]	2003	India	NM	Hospital—CC	2 m–5 y	Both	Middle	RIA	70	15.36	NM
Filteau et al. [48]	2015	India	NM	Community—CS	Mean age 5 y	Both	Lower	RIA	902	13.08	9.2
Taru et al. [49]	2015	India	NM	Hospital—CC	NM	Both	NM	NM	50	7.25	6.29
Sreedharan et al. [50]	2018	India	NM	Hospital—CC	2–13 y	Both	NM	ELISA	109	30.1	23.42
Agarwal et al. [51]	2010	India	Urban	Hospital—CS	10 weeks	Both	Lower	RIA	97	10.47	6.74
Mathur et al. [52]	2016	India	NM	Hospital—RCT	NM	Both	NM	ECLIA	50	12.65	10
Kumar et al. [53]	2011	India	NM	Hospital—RCT	6 months	Both	NM	RIA	237	14.4	10.2
Marwaha et al. [54]	2011	India	NM	Hospital—CS	6 weeks	Both	Lower	RIA	342	8.92	4.2
Agrawal et al. [55]	2019	India	Both	Hospital—CC	3 d–21 d	Both	NM	CLIA	50	14.88	7.2
Shukla et al. [56]	2016	India	NM	Hospital—CS	< 20 y	Both	NM	ECLIA	73	14.115	7.725
Schulze et al. [57]	2014	Nepal	Rural	Community—RCT	6–8 y	Both	NM	CIA	1000	26.52	7.4
Haugen et al. [58]	2016	Nepal	Urban	Community—CS	1–12 m	Both	NM	LCMS	466	32.8	8.56
Avagyan et al. [59]	2015	Nepal	Rural	Community—CS	1 y–5 y	Both	NM	LCMS	280	12.6	4.8
Marasinghe et al. [60]	2015	Sri Lanka	Urban	Community—CS	2–5 y	Both	NM	CLIA	340	23.5	8.97
Hettiarachchi et al. [61]	2010	Sri Lanka	NM	Hospital—CS	3–5 y	Both	NM	IRMA	248	33.71	14.1
Hettiarachchi et al. [62]	2011	Sri Lanka	NM	Hospital—CS	3–5 y	Both	NM	IRMA	105	24.78	0.66
Anwar et al. [63]	2015	Pakistan	Both	Community—CS	NM	Both	Lower	CLIA	227	9.884	7.334
Karim et al. [64]	2010	Pakistan	NM	Hospital—CS	NM	Both	Both	NM	50	20.4	10.99
Ahmed et al. [65]	2015	Bangladesh	Urban	Community—CS	0.5–2 y	Both	Both	EIA	913	21.86	16.67
Holland et al. [66]	2007	Afghanistan	NM	Community—CS	0.5–5 y	Both	NM	HPLC	107	5	NM

RCT = Randomized control trial, CC = Case–control, CS = Cross-sectional, CIA = Commercial immunoassay, RIA = Radio immune assay, ELISA = Enzyme-linked immune sorbent assay, CLIA = Chemiluminescence immunoassay, CMIA = Chemiluminescent microparticle immune assay, IRMA = Immunoradiometric immunoassay, HPLC = High-performance liquid chromatography, LCMS = Liquid chromatography tandem mass spectroscopy, EIA = Enzyme Immunoassay, ECLIA = Electrochemiluminescence Immunoassay, NM = Not mention, y = Year m = Month, d = day

*Belongs to a single study. This study was conducted on a population from two separate age groups (below-6 years and above-6 years) and therefore we segregated the sample size into two groups for our analysis

**Fig. 2:**
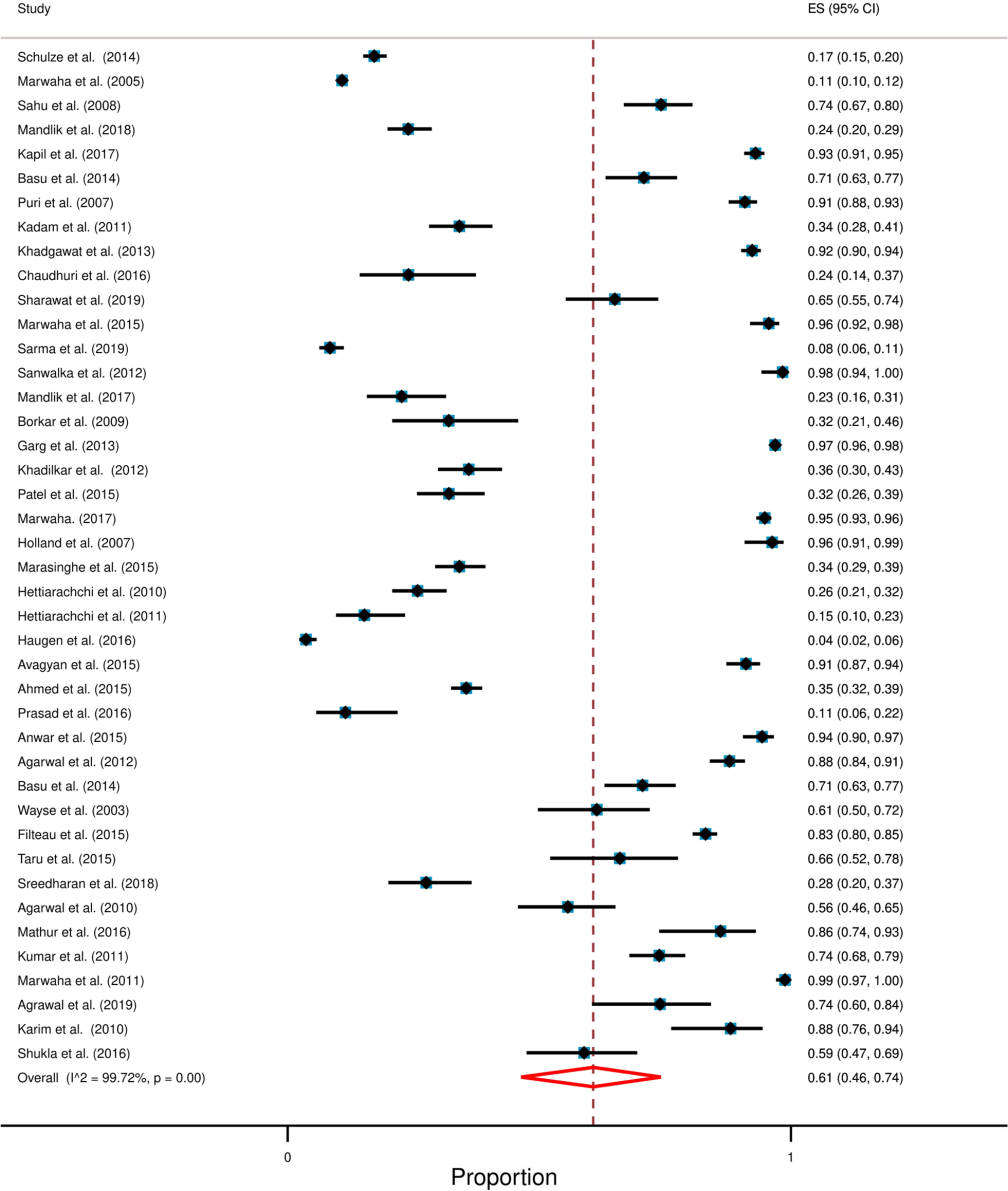
Forest plot displays the overall prevalence of hypovitaminosis D among South Asian children and adolescents. Each horizontal line of the forest plot represents an individual study and the box is plotted as prevalence for that study. Diamond at the bottom represents overall polled prevalence when all the individual studies are combined and averaged. The horizontal points of the diamond represent the limit of 95% confidence interval

Studies selected in this systematic review consisted of 18,233 participants. While most of these studies reported serum vitamin D levels of children up to 18 years of age, two studies included participants of up to 20 years [27, 56]. The overall pooled prevalence of hypovitaminosis D was 61% [95% CI: 46% to 71%] with a high degree of heterogeneity (*I*^2^ = 99.72%; *p* < 0.0001). Figure 2 shows the overall forest plot about the prevalence of hypovitaminosis D in South Asia.

Prevalence of hypovitaminosis D and the average level of serum vitamin D was mentioned in all studies; prevalence ranged from 8 to 96% and average ranged from 5 ng/mL to 34 ng/mL (for the individual studies). The weighted mean level of serum vitamin D was 15.48 ng/mL and the weighted standard deviation (weighted SD) was 7.49 ng/mL.

### Effect of geographical location on the prevalence of hypovitaminosis D

**Fig. 3:**
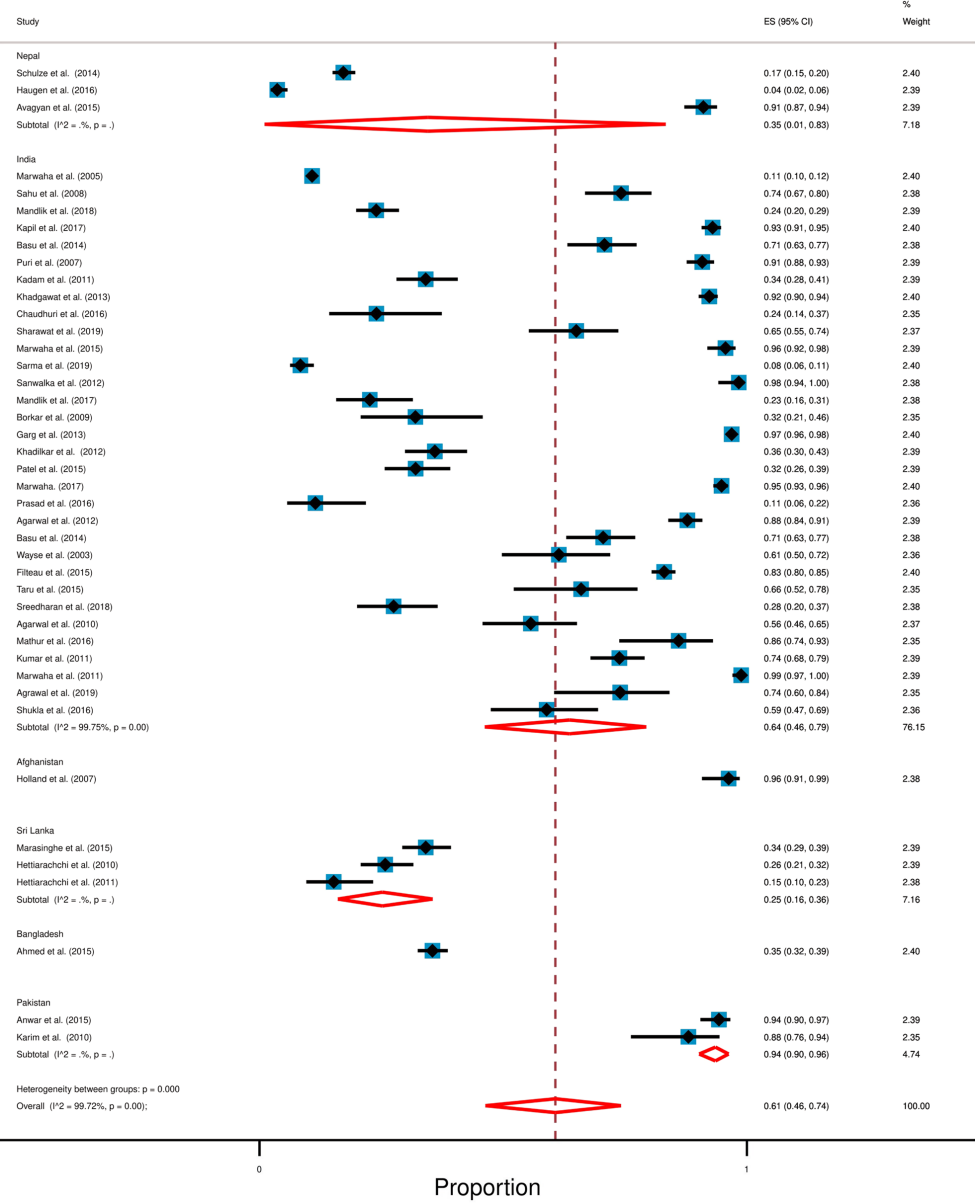
Forest plot displays the country-wise prevalence of hypovitaminosis D among South Asian children and adolescents. In this forest pot, all the diamonds, except the last one (overall pooled prevalence) represent polled prevalence for the individual country; (1) Nepal, (2) India, (3) Afghanistan, (4) Sri Lanka, (5) Bangladesh, (6) Pakistan. Each horizontal line of the forest plot represents an individual study and the box is plotted as prevalence for that study. The horizontal points of the diamond represent the limit of 95% confidence interval

We found studies following our inclusion criteria from 6 out of 8 South Asian countries. No studies were found from Bhutan and Maldives. A summary table shows the country-wise result (Table 2). We found that Afghanistan has the highest and Sri Lanka has the lowest prevalence of hypovitaminosis D in South Asia. The Forest plot shows the country-wise prevalence of hypovitaminosis D (Fig. 3). A bar diagram shows the weighted mean level of vitamin D among South Asian children (Additional file 1: Figure S1).

**Table 2:** Result in accordance to country

Country	Study found	Total participants	Weighted mean level of vitamin D (Weighted standard deviation)	Prevalence of hypovitaminosis
India [26–56]	31	14,497	13.40 ng/mL (6.61)	64% [95% CI: 46% to 79%]
Nepal [57–59]	3	1746	25.96 ng/mL (7.29)	35% [95% CI: 1% to 83%.]
Sri Lanka [60–62]	3	693	27.34 ng/mL (9.54)	25% [95% CI: 16% to 36%]
Pakistan [63, 64]	2	277	11.78 ng/mL (7.99)	94% [95% CI: 90% to 96%]
Afghanistan [65]	1	107	5 ng/mL*	96.2% [95% CI: 91% to 99%]
Bangladesh [66]	1	913	21.86 ng/mL (16.67)	35.48% [95% CI: 32% to 39%]

*Standard deviation was not mentioned in this study

### India

We found thirty-one studies from India with a total of 14,497 participants [26–56]. The weighted mean level of hypovitaminosis D for study participants was 13.40 ng/mL (SD 6.61 ng/mL) and random-effect meta-analysis showed that the pooled prevalence of hypovitaminosis D was 64% [95% CI: 46% to 79%] with a high level of heterogeneity (*I*^2^ = 99.75%; *p* < 0.0001).

### Nepal

There were three studies from Nepal which consisted of 1746 participants [57–59]. The random-effect meta-analysis pointed that the prevalence of hypovitaminosis D was 35% [95% CI: 1% to 83%.] and the weighted mean level of serum vitamin D for study participants was 25.96 ng/mL (weighted SD 7.29 ng/mL).

### Sri Lanka

In Sri Lanka, there were three studies comprised of 693 participants [60–62]. The weighted mean level of serum vitamin D for study participants was 27.34 ng/mL (weighted SD 9.54 ng/mL) and random-effect meta-analysis showed that the weighted pooled prevalence of hypovitaminosis D was 25% [95% CI: 16% to 36%].

### Pakistan

We found two studies from Pakistan [63, 64] which together consisted of 277 participants and the random-effect meta-analysis showed that 94% [95% CI: 90% to 96%] of participants were hypovitaminosis D with 11.78 ng/mL weighted mean level of serum vitamin D (weighted SD 7.99 ng/mL).

### Bangladesh

There was only one study from Bangladesh [65] which included 913 participants with 21.86 ng/mL mean level of serum vitamin D and 35.48% [95% CI: 32% to 39%] of them had hypovitaminosis D.

### Afghanistan

We found a single study from Afghanistan [66] which comprised 107 participants and the study result revealed 96.2% [95% CI: 91% to 99%] of them had hypovitaminosis D with 5 ng/mL mean level of serum vitamin D.

### Effect of gender on prevalence of hypovitaminosis D

**Fig. 4:**
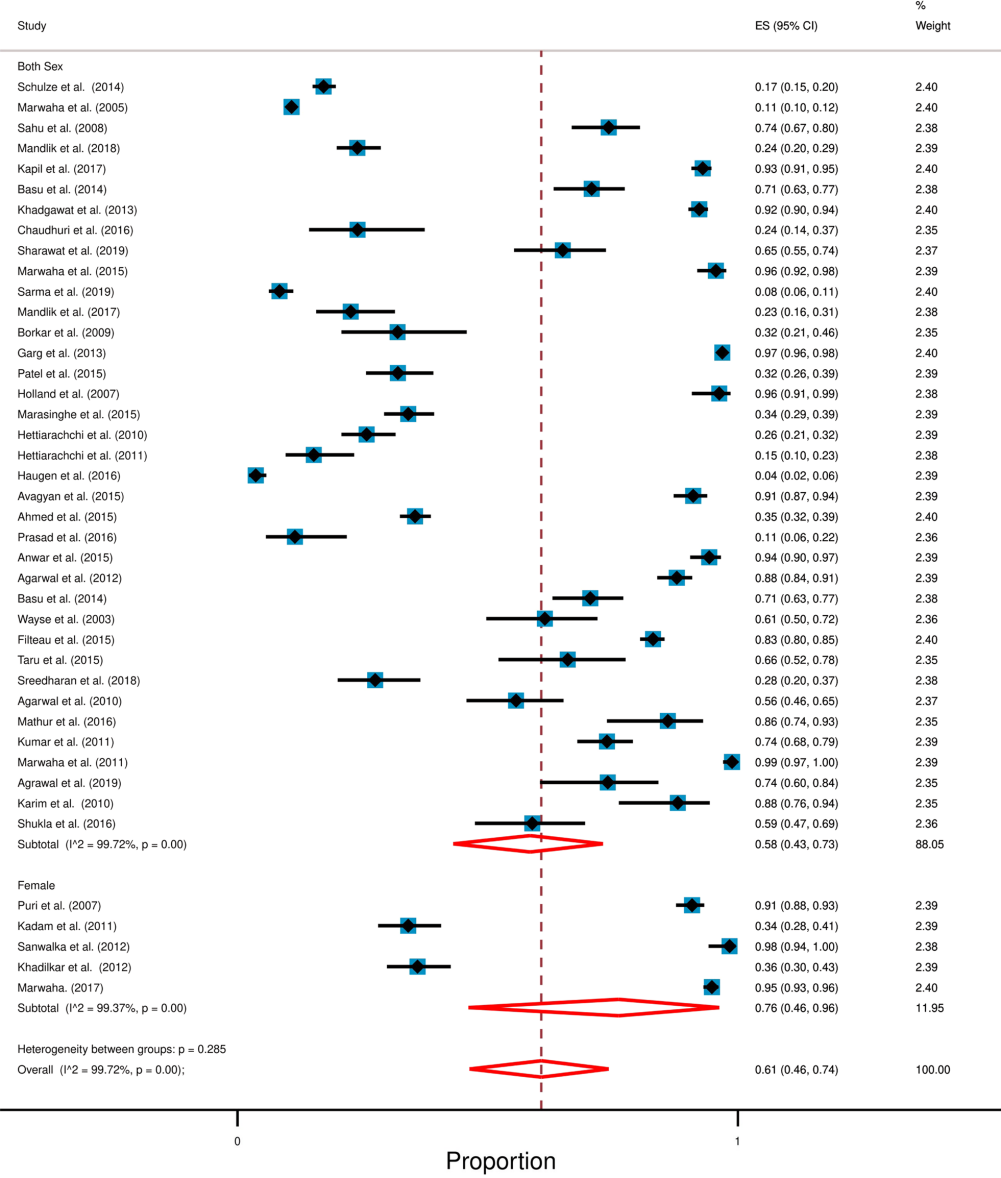
Forest plot following gender for the prevalence of hypovitaminosis D among South Asian children and adolescents. In this forest plot, all the diamonds except the last one (overall pooled prevalence) represent polled prevalence following gender. Here are two categories; studies that represent participants from both gender and the only female. Each horizontal line of the forest plot represents an individual study and the box is plotted as prevalence for that study. The horizontal points of the diamond represent the limit of 95% confidence interval

All of the studies we found were conducted either on both genders or only among female children and adolescents. A summary table shows gender-wise results (Table 3). We categorized this section into two parts. Studies which included participants from both gender and studies which consider only female children as their participants. Overall study result shows high degree of heterogeneity (*I*^2^ =  > 99%; *p* < 0.0001). Figure 4 shows the gender-wise forest plot.

**Table 3:** Result following gender

Gender	Number of studies	Total participants	Range of average vitamin D level (standard deviation)	Prevalence of hypovitaminosis
Both [26–30, 33–37, 39–41, 43, 45–66]	36	16,434	5 to 34 ng/mL (1 to 23)	58% [95% CI: 43% to 73%]
Female only [31, 32, 38, 42, 44]	5	1799	9 to 25 ng/mL (4 to 11)	76% [95% CI: 46% to 96%]

### Studies included participants from both gender

We found 36 out of 41 studies which included participants from both genders and among these studies, 26 studies were conducted in India, 2 studies in Pakistan, 3 studies in Nepal, 3 studies in Sri Lanka, and one study from Bangladesh and Afghanistan each [26–30, 33–37, 39–41, 43, 45–66]. Together, these studies comprised of 16,434 participants, and a random-effect meta-analysis indicated that 58% [95% CI: 43% to 73%] of study participants were hypovitaminosis D with a high degree of heterogeneity (*I*^2^ = 98.72%; *p* < 0.0001) The mean level of serum vitamin D ranged from 5 ng/mL to 34 ng/mL (for the individual studies) among study participants.

### Studies included only female participants

We found 5 out of 41 studies that included only female participants and all of these studies were conducted in India [31, 32, 38, 42, 44]. These studies together comprised 1799 participants and random-effect meta-analysis demonstrated that 76% [95% CI: 46% to 96%] of study participants had hypovitaminosis D with a high number of heterogeneity (*I*^2^ = 99.37%; *p* < 0.0001). The mean level of serum vitamin D among study participants ranged from 9 to 24 ng/mL.

### Prevalence of hypovitaminosis D for different age groups

**Fig. 5:**
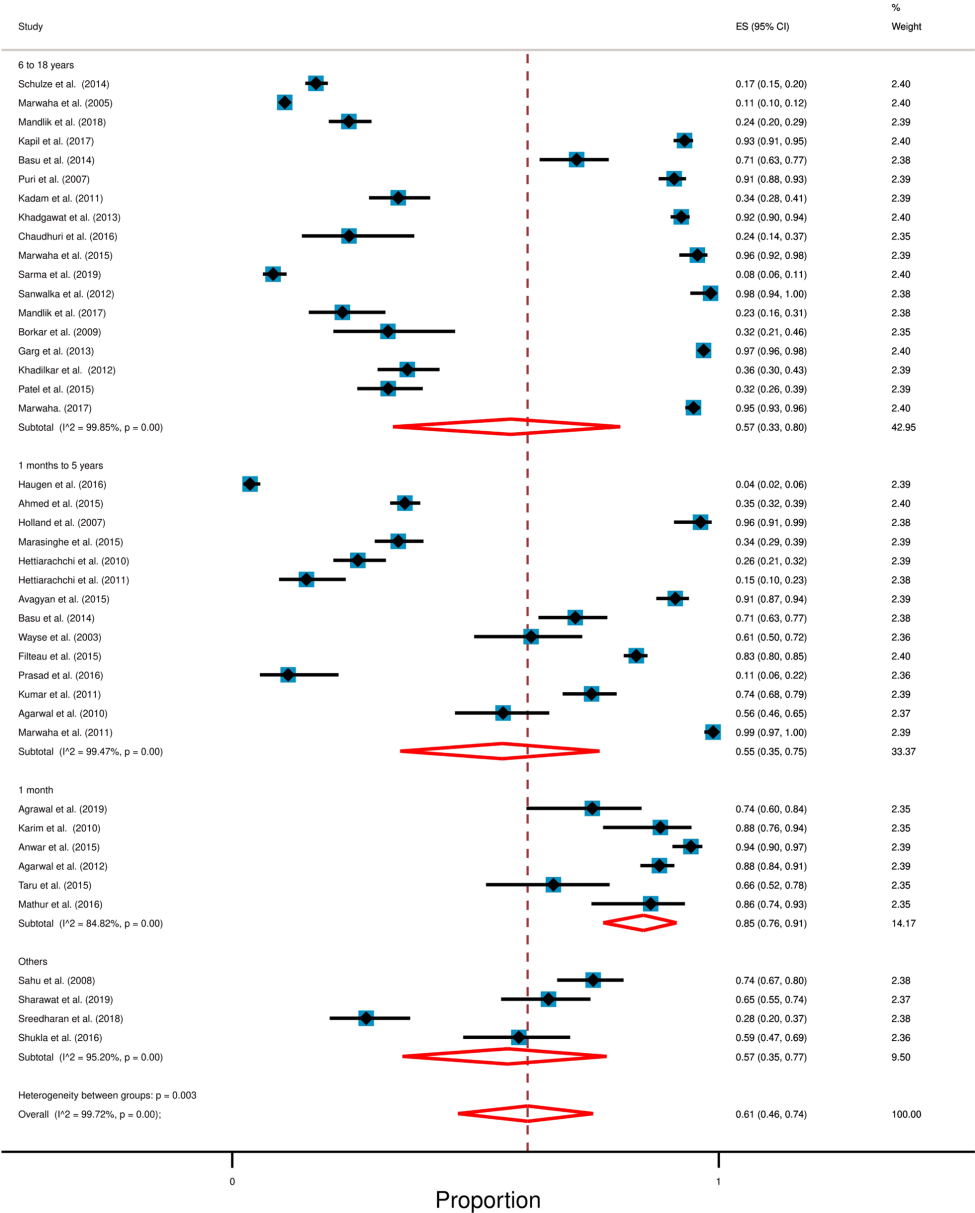
Forest plot following age for the prevalence of hypovitaminosis D among South Asian children and adolescents. In this forest plot, all the diamonds except the last one (overall pooled prevalence) represent polled prevalence following age. Here are four categories; studies; (1) 6–18 years of age (school) (2) < 5 years (preschool and infants), (3) up to 1-month (neonates), and (4) others. Each horizontal line of the forest plot represents an individual study and the box is plotted as prevalence for that study. The horizontal points of the diamond represent the limit of 95% confidence interval

In this section, we categorized study participants into four groups according to their age and these are 1 month (neonates), 1 month to 5 years (infants and preschool children), 6 to 18 years (school children), and others (< 20 years). A summary table shows the age-wise result (Table 4). We found out that, in South Asia, infants and preschool children have the lowest and neonates have the highest prevalence of hypovitaminosis D. Overall study result shows high degree of heterogeneity (*I*^2^ = 99.72%; *p* < 0.0001). Forest plot with further detail is available in Fig. 5.

**Table 4:** Result following age

Age	Category	Study found	Total participants	Range of average vitamin D level (standard deviation)	Prevalence of hypovitaminosis
1 month [46, 49, 52, 55, 63, 64]	Neonates	6	763	6 to 20 ng/mL (4 to 11)	85% [95% CI: 76% to 91%]
1 month–5 years [45, 47, 48, 51, 53, 54, 57–62, 65, 66]	Infants and preschool children	14	4324	5 to 34 ng/mL (1 to 17)	55% [95% CI: 35% to 75%]
6–18 years [26, 28–34, 36–44]	School children	17	12,709	6 to 26 ng/mL (1 to 17)	57% [95% CI: 33% to 80%]
2–20 years [27, 35, 50, 56]	Others	4	437	14 to 30 ng/mL (8 to 23)	57% [95% CI: 35% to 77%]

### School children (6–18 years)

We identified 17 out of 41 studies with individuals aged 6 to 18 years old. Only one of these studies was conducted in Nepal and the rest were conducted in India [26, 28–34, 36–44]. Together, these studies consisted of 12,709 participants and random-effect meta-analysis showed that 57% [95% CI: 33% to 80%] of study participants had hypovitaminosis D with a high degree of heterogeneity (*I*^2^ = 99.85%; *p* < 0.0001). The mean level of serum vitamin D among study participants ranged from 6.3 ng/mL to 26.52 ng/mL (for the individual studies).

### Infants and preschool children (1 month–5 years)

There were 14 out of 41 studies which included participants who were 1 month to 5 years of age and among these studies 7 were conducted in India, 3 in Sri Lanka, 2 in Nepal, and one study was conducted in Bangladesh and Afghanistan each [45, 47, 48, 51, 53, 54, 57–62, 65, 66]. Together, these studies consisted of 4324 participants with 5 ng/mL to 33.71 ng/mL mean serum level of vitamin D. Random-effect meta-analysis showed that 55% [95% CI: 35% to 75%] of study participants had hypovitaminosis D with a high degree of heterogeneity (*I*^2^ = 99.47%; *p* < 0.0001).

### Neonates (1 month)

We found 6 out of 41 studies which included participants who were up to 1 month in age. Among, these studies 4 were conducted in India and 2 in Pakistan [46, 49, 52, 55, 63, 64]. Together, these studies consisted of 763 participants and random-effect meta-analysis revealed that 85% [95% CI: 76% to 91%] of study participants had hypovitaminosis D with a high degree of heterogeneity (*I*^2^ = 84.82%; *p* < 0.0001). The mean level of serum vitamin D ranged from 6 ng/mL to 20 ng/mL among study participants.

### Others

In this group participants' age range was < 20 years. In total, we found 4 studies in this section, and all of these were conducted in India [27, 35, 50, 56]. These studies together consisted of 437 participants and random-effect meta-analysis showed that 57% [95% CI: 35% to 77%] of study participants had hypovitaminosis D with a high degree of heterogeneity (*I*^2^ = 95.20%; *p* < 0.0001). The average vitamin D level of study participants ranged from 14 to 30 ng/mL.

### Effect of study setting on the prevalence of hypovitaminosis D

**Fig. 6:**
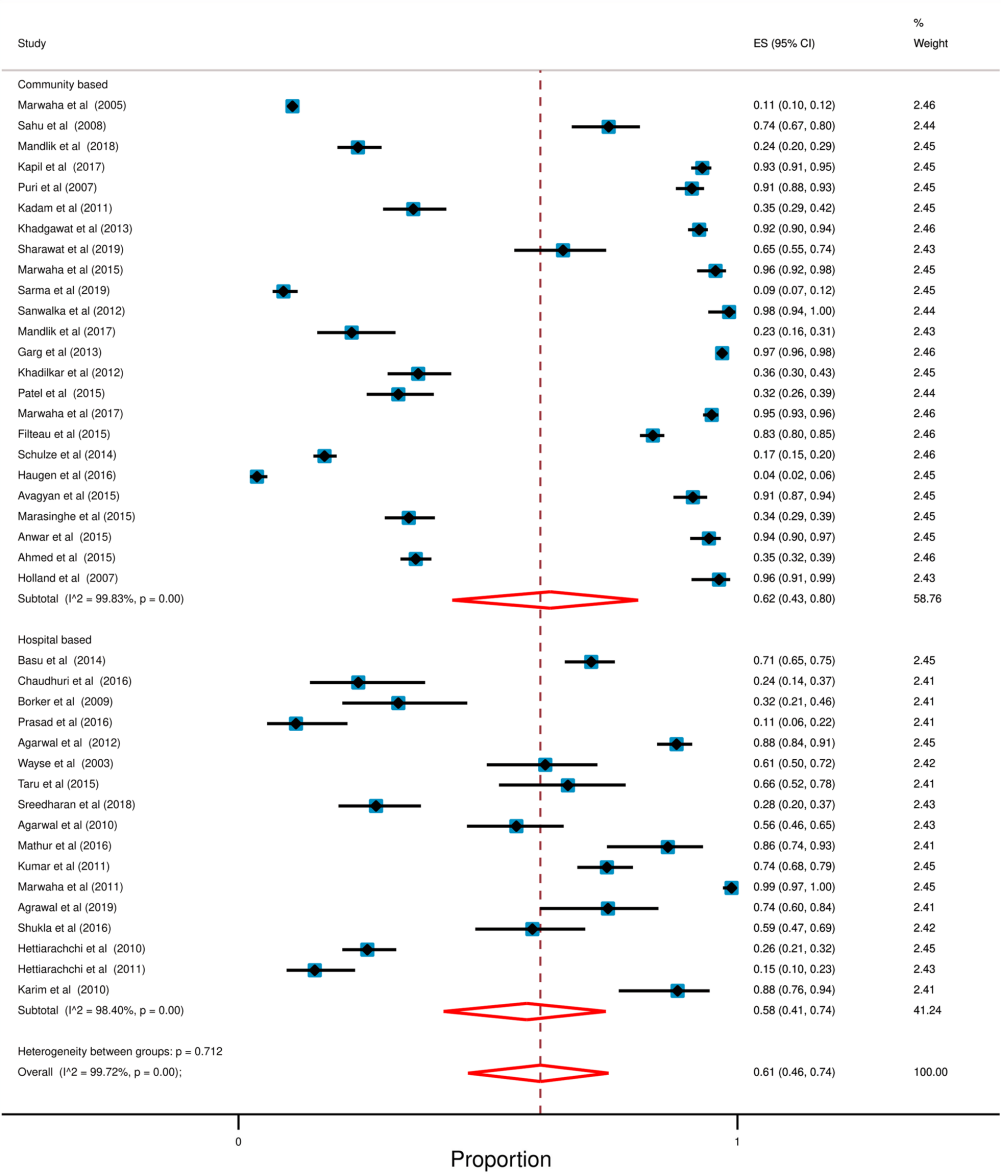
Forest plot in accordance with study setting for the prevalence of hypovitaminosis D. In this forest plot, all the diamonds except the last one (overall pooled prevalence) represent polled prevalence following study setting. Here are two categories; studies; (1) Community Based (2) Hospital Based. Each horizontal line of the forest plot represents an individual study and the box is plotted as prevalence for that study. The horizontal points of the diamond represent the limit of 95% confidence interval

Most of the selected studies were community-based (24 out of 41) and the rest were hospital-based (17 out of 41). Moreover, community-based study setting (62%; 95% CI: 43% to 80%) showed high prevalence of hypovitaminosis D in compared with hospital-based setting (58%; 95% CI: 41% to 74%). However, high degree of heterogeneity was observed in both of the study setting (Community: *I*^2^ = 99.83; *p* < 0.0001, Hospital: *I*^2^ = 98.40; *p* < 0.0001). Forest plot with additional information is presented in Fig. 6.

### Effect of lab methods on the prevalence of hypovitaminosis D

In our selected studies, the serum level of vitamin D was determined by using a variety of lab methods. Among these, Radioimmunoassay (RIA; 14 out of 41), Chemiluminescent Immunoassay (CLIA; 8 out of 41), and Enzyme-linked Immunosorbent assay (ELISA; 4 out of 41) were mostly used. Only two studies did not mention their procedure of vitamin D estimation [49, 64].

**Fig. 7:**
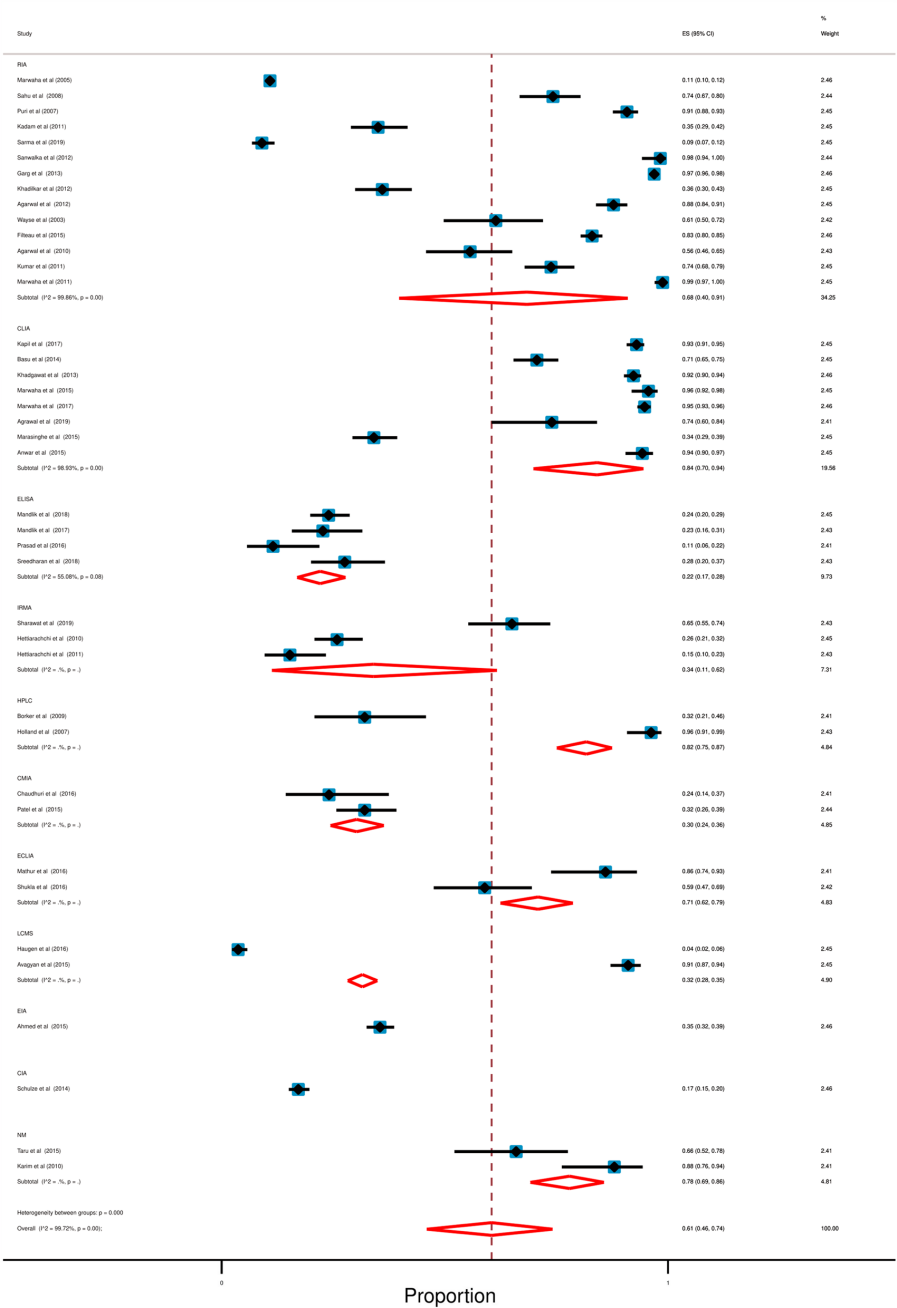
Forest plot in accordance with lab methods for the prevalence of hypovitaminosis D. In this forest plot, all the diamonds except the last one (overall pooled prevalence) represent polled prevalence following study setting. Each horizontal line of the forest plot represents an individual study and the box is plotted as prevalence for that study. The horizontal points of the diamond represent the limit of 95% confidence interval. Here are eleven categories; studies; (1) RIA or radio immune assay (2) CLIA or chemiluminescence immunoassay, (3) ELISA or enzyme-linked immune sorbent assay, (4) IRMA or immunoradiometric immunoassay, (5) HPLC or high-performance liquid chromatography, (6) CMIA or chemiluminescent microparticle immune assay, (7) ECLIA or electrochemiluminescence Immunoassay, (8) LCMS or liquid chromatography tandem mass spectroscopy, (9) EIA or enzyme immunoassay, (10) CIA or commercial immunoassay, (11) NM or not mention

However, ELISA demonstrated the lowest (22%; 95% CI: 17% to 28%) and CLIA (84%; 95% CI: 70% to 94%) showed the highest prevalence of hypovitaminosis D amid all of the measurement methods used. High degree of heterogeneity was also observed in this section (RIA: *I*^2^ = 99.86%; *p* < 0.0001, CLIA: *I*^2^ = 98.93%; *p* < 0.0001, ELISA: *I*^2^ = 55.08%; *p* = 0.08). Detail is accessible in forest plot (Fig. 7).

### Quality assessment

**Fig. 8:**
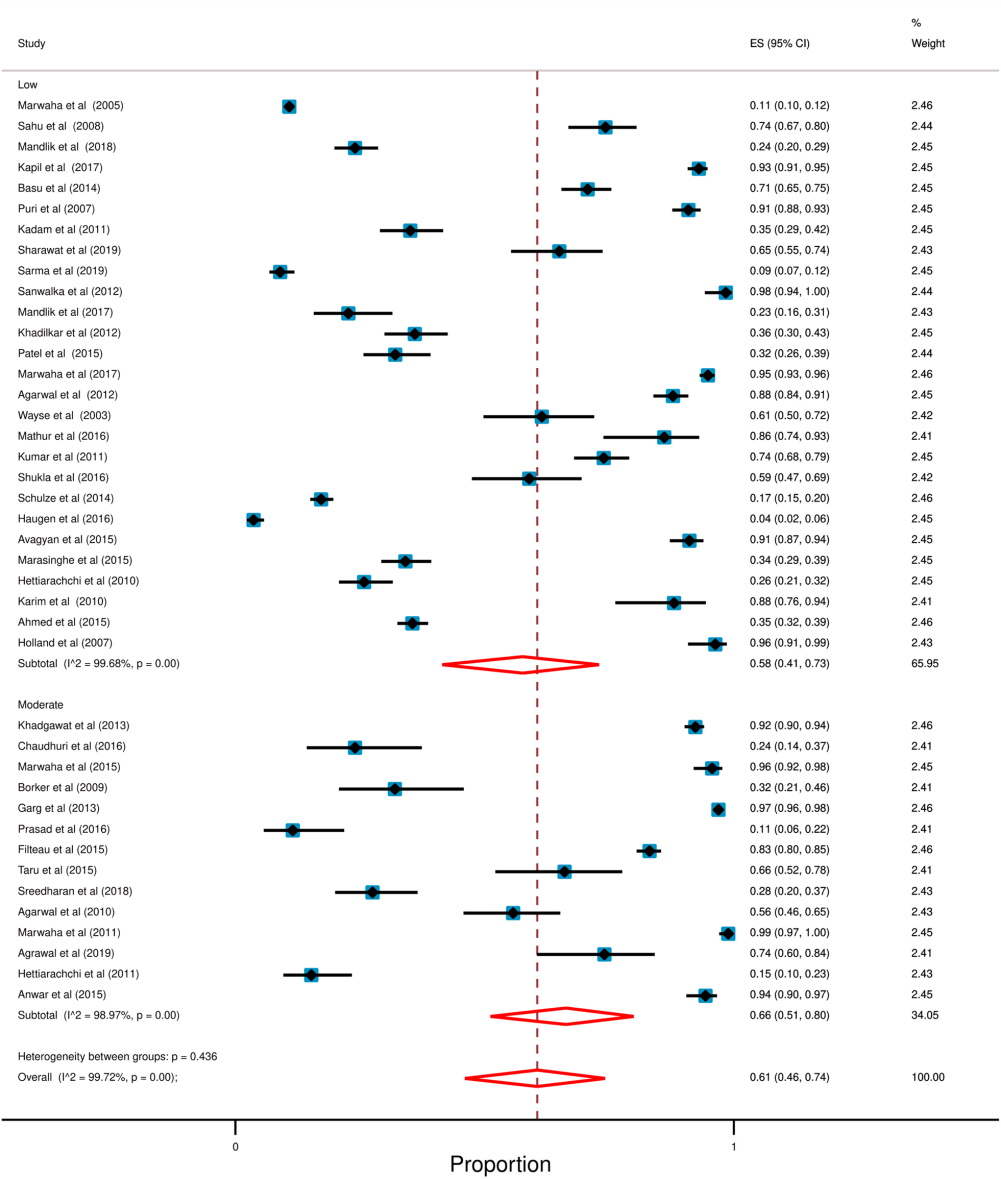
Forest plot in accordance with Risk of bias for the prevalence of hypovitaminosis D. In this forest plot, all the diamonds except the last one (overall pooled prevalence) represent polled prevalence following study setting. Here are two categories; studies; (1) Low (2) Moderate. Each horizontal line of the forest plot represents an individual study and the box is plotted as prevalence for that study. The horizontal points of the diamond represent the limit of 95% confidence interval

Among these selected studies no study was found with a high risk of bias, 14 studies have a moderate risk of bias and the rest contained a low risk of bias. The risk of Bias for selected studies is available in Additional file 1: Table S2. Furthermore, random-effect meta-analysis illustrated that, studies with moderate risk of bias (66%; 95% CI: 51% to 80%) has a high prevalence of hypovitaminosis D compared to studies with low risk of bias (58%; 95% CI: 41% to 73%). Furthermore, high degree of heterogeneity was also observed in risk of bias assessment (Low: *I*^2^ = 99.68%; *p* < 0.0001, Moderate: *I*^2^ = 98.97%; *p* < 0.0001). Figure 8 shows the forest plot.

### Publication bias

The presence of asymmetry and publication bias was indicated by the funnel plot. The Eggers test was found to be statistically insignificant, implying that small-study effects were not present (*p* = 0.74). The funnel plot is available in Additional file 1: Figure S2.

## Discussion

This study reveals that approximately 6 out of 10 South Asian children and adolescents (up to 18 years) could be affected with hypovitaminosis D (Fig. 2). Comparison of this hypovitaminosis D to the other parts of the world implies that this problem might be worse in South Asia compared to Africa (around 34%; taking < 20 ng/mL cut-off) [67]. We assume that hypovitaminosis D may be co-related to the high prevalence of some childhood health problems in this area. Indeed, this is supported by several reports suggesting a high burden of diseases that are associated with vitamin D deficiency like tuberculosis, obesity and overweight, type 1 diabetes, etc. among South Asian children and adolescents [4, 16–18, 68–71].

While high hypovitaminosis D was the highlight of this study, we also found significant amounts of heterogeneity in the overall result (*I*^2^ = 99.72%) and it can be assumed that geography might be acting as one of the significant variables. Because, vitamin D is synthesized naturally in our body when UVB from sunlight penetrates our skin and undergoes some physiological processes [2, 5]. This is likely since people living in tropical areas are exposed to more sunlight than those living in subtropical regions [2, 5]. Among the South Asian countries, Pakistan and Afghanistan are situated in a subtropical area, while Sri Lanka is situated in a tropical area [72]. In our study, we also found that Pakistan and Afghanistan had the highest prevalence of hypovitaminosis D in South Asia; 94% (nation-wide average of serum vitamin D 11.78 ng/mL) and 96% (nation-wide average of serum vitamin D 5 ng/mL), respectively. On the contrary, Sri Lanka has the lowest level of hypovitaminosis D in South Asia; 25% (nation-wide average of serum vitamin D 27.34 ng/mL). Our hypothesis is further strengthened by reports from other tropical and subtropical countries. For example, Brazil is located in a tropical region and according to published literature, only 28% of the Brazilian population had hypovitaminosis D, while Qatar, a subtropical country had a high prevalence (90%) of hypovitaminosis D [73, 74]. However, it must be noted that besides geographical variation, other variables could be affecting the inter-country variations.

Among the factors influencing the production of vitamin D, age, gender, and diet are notable [2, 5]. In our study, the age-wise analysis revealed that the prevalence of hypovitaminosis D is more among neonates (85%) than preschool (55%) and school-going children (57%) (Fig. 5). This may be associated with the high prevalence of vitamin D insufficiency (65%) among South Asian pregnant women which we have shown in one of our recent studies [75].

Most of the studies in South Asia enrolled participants from both genders. We did not find any study in South Asia that was conducted on males only. We found five studies that were conducted among females. Gender-wise comparison suggested that the studies that considered only females as participants showed a higher prevalence of hypovitaminosis D (76%; 95% CI: 46% to 96%) compared to those that considered participants from both genders (58%; 95% CI: 43% to 73%) (Fig. 4). This could be suggestive of the fact that female children in South Asia could be more affected with hypovitaminosis D compared to young males. This may be associated with cultural aspects and clothing practices of South Asian where females practice heavier clothing (traditional and religious full-body covering dresses like burqa, hijab, shari, salwar, kurta, etc.). A recent media report also pointed that these practices have increased dramatically in this region over the past three decades [76]. However, we also argue that early marriage (which concerning early pregnancy), poor education, and insufficient decision-making ability may have some combined effects resulting in such a high prevalence of hypovitaminosis D among South Asian girls. In this regard, lack of recommended dietary intake, early marriage, and lack of higher education among girls has been reported in this region [77, 78].

Moreover, South Asians are affected with malnutrition of nearly all forms [79, 80]. While the cod liver oil, mushrooms, egg yolk, fish; salmon, mackerel, tuna, and fortified foods; milk, yogurt, cheese, orange juice, etc. are commonly referred to as the primary dietary sources of vitamin D, the effects of variation in staple foods (rice and wheat) in the South Asian population is not yet fully understood [2, 5, 81, 82]. Therefore, we recommend further studies to understand the influence of staple foods on population-level serum vitamin D levels.

Furthermore, skin complexion may be another factor for such a high prevalence of hypovitaminosis D in this region. According to the Fitzpatrick scale, South Asians are quite darker in comparison to Europeans [83]. A large observational data suggested that the prevalence of hypovitaminosis D (taking < 20 ng/mL cut-off) is 40% among Europeans [84] which is much lower in comparison to what we have seen in this study for South Asia.

Additionally, our analysis also revealed that studies design with community-based setting (62%; 95% CI: 43% to 80%) has high prevalence of hypovitaminosis compared to hospital-based setting (58%; 95% CI: 41% to 74%). In accordance with iceberg phenomenon [85], these findings indicate that a substantial number of South Asian children are affected with hypovitaminosis D that is either subclinical, unreported, or concealed from view. Therefore, community-based study settings demonstrated such a high prevalence of hypovitaminosis in South Asian children.

The high burden of hypovitaminosis D among South Asian children is a public health concern that should be addressed as an emergency. Some researchers also proposed that deficiency of vitamin D should be treated as a pandemic in progress [84]. In this regard, a more recent study reported that vitamin D deficiency is also related to ‘cytokine storm’ (dramatic immune system overreaction) which causes COVID 19 patients more vulnerable [86]. However, we also proposed that further research is needed to check if our findings can be applied to a wider group of general populations. Moreover, our analysis revealed a high degree of heterogeneity, which we attempted to explain explicitly using various independent variables such as geolocation, gender, age, skin colour, and so on. However, we believe that further studies are required to fully comprehend the reasons behind such significant heterogeneity.

Despite the high prevalence of hypovitaminosis D among South Asian children and adolescents, we did not find any national-level nutritional guidelines or policies for vitamin D except in India [87]. For the prevention of hypovitaminosis D among children and adolescents, China, Japan, and South Korea have similar guidelines [88, 89]. Furthermore, supplementation and food fortification programmes have been proved to be successful in Europe to reduce hypovitaminosis D [90]. Moreover, some other challenges need to be addressed in South Asia. A negative attitude towards sunlight exposure can be a big challenge. It has been reported that Indian and Pakistani students had a lack of knowledge about vitamin D and a negative attitude towards sunlight exposure [91, 92]. This lack of knowledge, along with a negative attitude, could be a key factor behind staying away from sunlight which can lead to hypovitaminosis D. Therefore, keeping the socio-cultural aspects of the individual countries (e.g. clothing practice, skin complexion, and economic status) in consideration, awareness campaigns about the relationship between sunlight exposure as a source of vitamin D can be emphasized. Furthermore, active measures should be taken to expand the number of diagnostic tests for detecting the serum vitamin D level. To achieve this, increasing the number of tests centres, reducing the cost of testing the serum level of vitamin D by offering subsidies can also be considered by the governments in the South Asian region.

To the best of our knowledge, this is the first systematic review and meta-analysis to highlight the prevalence of hypovitaminosis D in South Asian children. But this study also has few limitations. We were unable to explore the effect of the season in our analysis due to data insufficiency—only three of our selected studies [36, 48, 65] provided season-specific data. Above 75% of our selected studies (31 out of 41) were conducted among Indian children. In this regard, the population of India is also disproportionately higher compared to the other South Asian countries. We did not find any studies from Maldives and Bhutan. So, we could not calculate the prevalence of hypovitaminosis D and the weighted mean level of serum vitamin D for these countries. Moreover, more than 60% of our selected studies did not mention demographic area (urban vs rural) and socioeconomic status (high income vs low income) for their study populations. Therefore, we were unable to find any correlations between hypovitaminosis D and these factors. Furthermore, multiple different methods were used in different studies (Table 1) to assay the serum level of vitamin D, which might have introduced some degree of assay bias. However, this limitation is inherent for studies like ours and was indeed unavoidable. Another limitation is that, since, the definition of children and adolescents were not uniform [93], and that many of our selected studies did not provide age-wise data, we could not perform the subgroup analysis for children and adolescents as separate age groups. Furthermore, vitamin D assessments are often expensive for South Asian children and adolescents due to their poor economic circumstances. So, it is possible that the children and adolescents who were enrolled in at least some studies or who participated in a study or a trial that included assessment of vitamin D were indeed suspected of deficiency/insufficiency of this vitamin. We assume that this is another inherent yet unavoidable limitation of our study. Therefore, we recommend that the readers should exercise caution before generalizing the extent of hypovitaminosis D in South Asia.

## Conclusions

Out study unveiled that around six out of ten South Asian children and adolescents could be suffering from hypovitaminosis D. These findings have generated evidence of the actual population-level data that underscores the urgency of prioritizing the mitigation strategies. The subgroup analyses have resulted in several hypotheses to explain the observed heterogeneity of hypovitaminosis D among different countries, age groups, and genders. While this systematic review focused on South Asian children, the knowledge and insight generated from this study can be applied to other regions and countries with comparable geographical and socio-cultural aspects.

## Supplementary Information

**Additional file 1: Figure S1**. Comparative weighted mean of serum D levels among the South Asian countries. The error bars represent weighted standard deviation (except for Afghanistan from where only one study was reported).

**Additional file 2: Figure S2**. Funnel plot for weighted mean values of serum vitamin D levels reported by the studies included in this systematic review (where a single dot represents the measurement). High number of observations falling outside the expected range on both sides indicate high heterogenity of reported serum vitamin D levels in South Asia.

## Data Availability

Only aggregated summaries of the data are provided in this manuscript. However, all data generated in this study can be made publicly available on request. Please contact the corresponding author for any kind of data request.
