# Supplementary material for: High burden of hypovitaminosis D among the children and adolescents in South Asia: a systematic review and meta-analysis
Source: J Health Popul Nutr. 2022 Mar 17;41:10. doi: 10.1186/s41043-022-00287-w (PMC8929474; doi:10.1186/s41043-022-00287-w)
Supplement: Supplementary file 1 — Additional file 1: Figure S1. Comparative weighted mean of serum D levels among the South Asian countries. The error bars represent weighted standard deviation (except for Afghanistan from where only one study was reported). [file 41043_2022_287_MOESM1_ESM.docx]

**Table S1:** Search strategy, original MeSH terms used in this study**.**

| **PubMed** | **SCOPUS** | **Google Scholar** |
| --- | --- | --- |
| (“Vitamin D Deficiency” [MeSH]) AND Bangladesh [MeSH] AND Children [MeSH]  (“Vitamin D”[MeSH]) AND Bangladesh[MeSH] AND Children [MeSH]  (“Vitamin D”[MeSH]) AND Bangladesh[MeSH] AND Prevalence [MeSH] AND Children [MeSH] | (INDEXTERMS(“Vitamin D Deficiency”) AND INDEXTERMS(BANGLADESH) AND INDEXTERMS(Children)) OR (TITLE-ABS-KEY(“Vitamin D”) AND TITLE-ABS-KEY(BANGLADESH) AND TITLE-ABS-KEY(Children)) OR INDEXTERMS(prevalence)) AND (TITLE-ABS-KEY(“Vitamin D”) AND TITLE-ABS-KEY(BANGLADESH) AND TITLE-ABS-KEY(Children)) | ((“Vitamin D”) OR (“Vitamin D3”)) AND ((prevalence) OR (deficiency)) AND (Bangladesh) AND Children |
| (“Vitamin D Deficiency”[MeSH]) AND Bangladesh[MeSH] AND Neonates [MeSH]  (“Vitamin D”[MeSH]) AND Bangladesh[MeSH] AND Prevalence [MeSH] AND Children [MeSH]  (“Vitamin D”[MeSH]) AND Bangladesh[MeSH] AND Neonates [MeSH] | (INDEXTERMS(“Vitamin D Deficiency”) AND INDEXTERMS(BANGLADESH) AND INDEXTERMS(Neonates)) OR (TITLE-ABS-KEY(“Vitamin D”) AND TITLE-ABS-KEY(BANGLADESH) AND TITLE-ABS-KEY(Neonates)) OR INDEXTERMS(prevalence)) AND (TITLE-ABS-KEY(“Vitamin D”) AND TITLE-ABS-KEY(BANGLADESH) AND TITLE-ABS-KEY(Neonates)) | ((“Vitamin D”) OR (“Vitamin D3”)) AND ((prevalence) OR (deficiency)) AND (Bangladesh) AND Newborn |
| (“Vitamin D”[MeSH]) AND Bangladesh[MeSH] AND Prevalence [MeSH] AND Neonates [MeSH]  (“Vitamin D Deficiency”[MeSH]) AND Bangladesh[MeSH] AND Infants[MeSH]  (“Vitamin D”[MeSH]) AND Bangladesh[MeSH] AND Infants [MeSH]  (“Vitamin D”[MeSH]) AND Bangladesh[MeSH] AND Prevalence [MeSH] AND Newborn [MeSH] | (INDEXTERMS(“Vitamin D Deficiency”) AND INDEXTERMS(BANGLADESH) AND INDEXTERMS(Infants)) OR (TITLE-ABS-KEY(“Vitamin D”) AND TITLE-ABS-KEY(BANGLADESH) AND TITLE-ABS-KEY(Infants)) OR INDEXTERMS(prevalence)) AND (TITLE-ABS-KEY(“Vitamin D”) AND TITLE-ABS-KEY(BANGLADESH) AND TITLE-ABS-KEY(Infants)) | ((“Vitamin D”) OR (“Vitamin D3”)) AND ((prevalence) OR (deficiency)) AND (Bangladesh) AND Neonates |
| (“Vitamin D Deficiency”[MeSH]) AND Bangladesh[MeSH] AND Newborn [MeSH] (“Vitamin D”[MeSH]) AND Bangladesh[MeSH] AND Newborn [MeSH] | (INDEXTERMS (“Vitamin D Deficiency”) AND INDEXTERMS(BANGLADESH) AND INDEXTERMS(Pediatrics)) OR (TITLE-ABS-KEY (“Vitamin D”) AND TITLE-ABS- KEY(BANGLADESH) AND | ((“Vitamin D”) OR (“Vitamin D3”)) AND ((prevalence) OR (deficiency)) AND (Bangladesh) AND Infants |
| **PubMed** | **SCOPUS** | **Google Scholar** |
| (“Vitamin D”[MeSH]) AND Bangladesh[MeSH] AND Prevalence [MeSH] AND Pediatrics [MeSH]  (“Vitamin D”[MeSH]) AND Bangladesh[MeSH] AND Prevalence [MeSH] AND Adolescents [MeSH] | (INDEXTERMS (“Vitamin D Deficiency”) AND INDEXTERMS(BANGLADESH) AND INDEXTERMS(Pediatrics)) OR (TITLE-ABS-KEY (“Vitamin D”) AND TITLE-ABS-KEY(BANGLADESH) AND TITLE-ABS-KEY(Pediatrics)) OR INDEXTERMS (prevalence)) AND (TITLE-ABS-KEY (“Vitamin D”) AND TITLE-ABS-KEY(BANGLADESH) AND TITLE-ABS-KEY(Pediatrics)) | ((“Vitamin D”) OR (“Vitamin D3”)) AND ((prevalence) OR (deficiency)) AND (Bangladesh) AND Pediatrics |
| (“Vitamin D Deficiency”[MeSH]) AND Bangladesh[MeSH] AND Adolescents [MeSH]  (“Vitamin D”[MeSH]) AND Bangladesh[MeSH] AND Adolescents [MeSH] | (INDEXTERMS(“Vitamin D Deficiency”) AND INDEXTERMS(BANGLADESH) AND INDEXTERMS(Newborn)) OR (TITLE-ABS-KEY(“Vitamin D”) AND TITLE-ABS-KEY(BANGLADESH) AND TITLE-ABS-KEY(Newborn)) OR INDEXTERMS(prevalence)) AND (TITLE-ABS-KEY(“Vitamin D”) AND TITLE-ABS-KEY(BANGLADESH) AND TITLE-ABS-KEY(Newborn)) | ((“Vitamin D”) OR (“Vitamin D3”)) AND ((prevalence) OR (deficiency)) AND (Bangladesh) AND Adolescents |
|  | (INDEXTERMS(“Vitamin D Deficiency”) AND INDEXTERMS(BANGLADESH) AND INDEXTERMS(Adolescents)) OR (TITLE-ABS-KEY(“Vitamin D”) AND TITLE-ABS-KEY(BANGLADESH) AND TITLE-ABS-KEY(Adolescents)) OR INDEXTERMS(prevalence)) AND (TITLE-ABS-KEY(“Vitamin D”) AND TITLE-ABS-KEY(BANGLADESH) AND TITLE-ABS-KEY(Adolescents)) |  |

**By replacing the country name (e.g., India, Pakistan Sri Lanka etc.) same types of search terms were used for seven other South Asian countries to complete the search procedures.**

**Table S2:** Quality assessment of selected studies (Q1, Q2, Q3……Q10 denotes ten parameters described by Hoy et al for-quality assessment)

| **Authors** | Q1 | Q2 | Q3 | Q4 | Q5 | Q6 | Q7 | Q8 | Q9 | Q10 | Total score | Quality |
| --- | --- | --- | --- | --- | --- | --- | --- | --- | --- | --- | --- | --- |
| Marwaha et al (26) | 0 | 0 | 0 | 0 | 0 | 0 | 0 | 0 | 1 | 0 | 1 | Low |
| Sahu et al (27) | 1 | 0 | 0 | 0 | 0 | 1 | 0 | 0 | 0 | 0 | 2 | Low |
| Mandlik et al(28) | 1 | 0 | 0 | 0 | 0 | 0 | 0 | 0 | 1 | 0 | 2 | Low |
| Kapil et al (29) | 0 | 0 | 0 | 0 | 0 | 0 | 0 | 0 | 1 | 0 | 1 | Low |
| Basu et al (30) | 1 | 1 | 1 | 0 | 0 | 0 | 0 | 0 | 0 | 0 | 3 | Low |
| Puri et al (31) | 0 | 0 | 0 | 0 | 0 | 0 | 0 | 0 | 1 | 0 | 1 | Low |
| Kadam et al (32) | 1 | 1 | 0 | 0 | 0 | 0 | 0 | 0 | 1 | 0 | 3 | Low |
| Khadgawat et al (33) | 1 | 1 | 1 | 0 | 0 | 0 | 0 | 0 | 1 | 0 | 4 | Moderate |
| Chaudhuri et al(34) | 1 | 1 | 1 | 0 | 0 | 0 | 0 | 0 | 1 | 0 | 4 | Moderate |
| Sharawat et al(35) | 1 | 0 | 0 | 0 | 0 | 0 | 0 | 0 | 1 | 0 | 2 | Low |
| Marwaha et al(36) | 1 | 1 | 1 | 0 | 0 | 0 | 0 | 0 | 1 | 0 | 4 | Moderate |
| Sarma et al(37) | 1 | 1 | 0 | 0 | 0 | 0 | 0 | 0 | 1 | 0 | 3 | Low |
| Sanwalka et al(38) | 0 | 0 | 0 | 0 | 0 | 0 | 0 | 0 | 1 | 0 | 1 | Low |
| Mandlik et al(39) | 1 | 0 | 0 | 0 | 0 | 0 | 0 | 0 | 1 | 0 | 2 | Low |
| Borker et al(40) | 1 | 1 | 1 | 0 | 0 | 0 | 0 | 0 | 1 | 0 | 4 | Moderate |
| Garg et al(41) | 1 | 1 | 1 | 0 | 0 | 0 | 0 | 0 | 1 | 0 | 4 | Moderate |
| Khadilkar et al (42) | 1 | 0 | 0 | 0 | 0 | 0 | 0 | 0 | 1 | 0 | 2 | Low |
| Patel et al(43) | 0 | 0 | 0 | 0 | 0 | 0 | 0 | 0 | 1 | 0 | 1 | Low |
| Marwaha et al(44) | 0 | 0 | 0 | 0 | 0 | 0 | 0 | 0 | 1 | 0 | 1 | Low |
| Prasad et al(45) | 1 | 1 | 1 | 0 | 0 | 0 | 0 | 0 | 1 | 1 | 5 | Moderate |
| Agarwal et al(46) | 1 | 1 | 1 | 0 | 0 | 0 | 0 | 0 | 0 | 0 | 3 | Low |
| Wayse et al(47) | 1 | 1 | 1 | 0 | 0 | 0 | 0 | 0 | 0 | 0 | 3 | Low |
| Filteau et al (48) | 0 | 1 | 1 | 0 | 0 | 0 | 1 | 0 | 1 | 1 | 5 | Moderate |
| Taru et al (49) | 1 | 1 | 1 | 0 | 0 | 0 | 0 | 0 | 1 | 0 | 4 | Moderate |
| Sreedharan et al (50) | 1 | 1 | 1 | 0 | 0 | 0 | 0 | 0 | 1 | 1 | 5 | Moderate |
| Agarwal et al (51) | 1 | 1 | 1 | 0 | 0 | 0 | 0 | 0 | 1 | 0 | 4 | Moderate |
| Mathur et al (52) | 1 | 0 | 0 | 0 | 0 | 0 | 0 | 0 | 0 | 0 | 1 | Low |
| Kumar et al (53) | 1 | 0 | 0 | 0 | 0 | 0 | 0 | 0 | 0 | 0 | 1 | Low |
| Marwaha et al(54) | 1 | 1 | 1 | 0 | 0 | 0 | 1 | 0 | 1 | 1 | 6 | Moderate |
| Agrawal et al(55) | 1 | 1 | 1 | 0 | 0 | 0 | 0 | 0 | 1 | 1 | 5 | Moderate |
| Shukla et al(56) | 0 | 1 | 1 | 0 | 0 | 0 | 1 | 0 | 0 | 0 | 3 | Low |
| Schulze et al (57) | 0 | 0 | 0 | 0 | 0 | 0 | 0 | 0 | 1 | 0 | 1 | Low |
| Haugen et al(58) | 0 | 0 | 0 | 0 | 0 | 0 | 0 | 0 | 1 | 1 | 2 | Low |
| Avagyan et al(59) | 1 | 0 | 0 | 0 | 0 | 0 | 0 | 0 | 1 | 0 | 2 | Low |
| Marasinghe et al(60) | 0 | 0 | 0 | 0 | 0 | 0 | 0 | 0 | 1 | 0 | 1 | Low |
| Hettiarachchi et al(61) | 0 | 0 | 0 | 0 | 0 | 0 | 0 | 0 | 1 | 1 | 2 | Low |
| Hettiarachchi et al(62) | 1 | 1 | 0 | 0 | 0 | 0 | 0 | 0 | 1 | 1 | 4 | Moderate |
| Anwar et al(63) | 1 | 1 | 1 | 0 | 0 | 0 | 0 | 0 | 1 | 0 | 4 | Moderate |
| Karim et al (64) | 0 | 0 | 0 | 0 | 0 | 0 | 0 | 0 | 1 | 0 | 1 | Low |
| Ahmed et al(65) | 1 | 0 | 0 | 0 | 0 | 0 | 0 | 0 | 0 | 0 | 1 | Low |
| Holland et al(66) | 0 | 0 | 0 | 0 | 0 | 0 | 0 | 0 | 1 | 1 | 2 | Low |

**
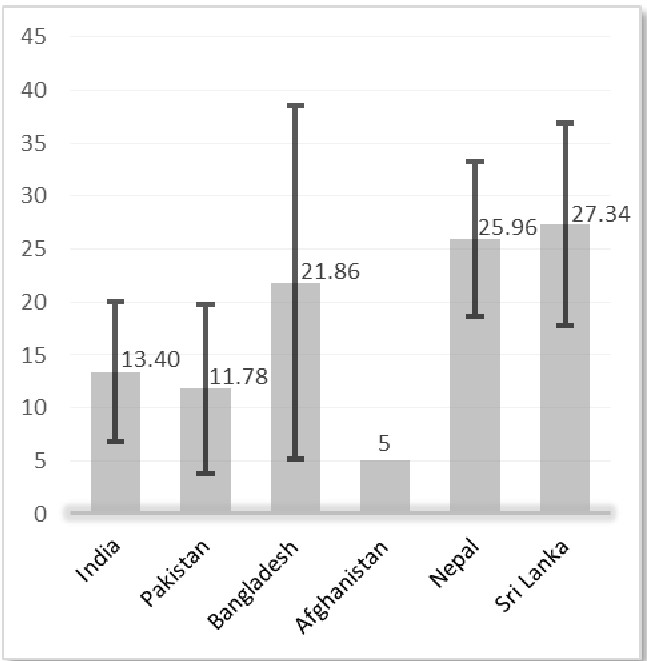
**

**Figure S1:** Weighted mean level of serum vitamin D (ng/mL) among South Asian children (on the Y-axis). X-axis represent the name of the South Asian countries. Error bars represent the weighted standard deviations.


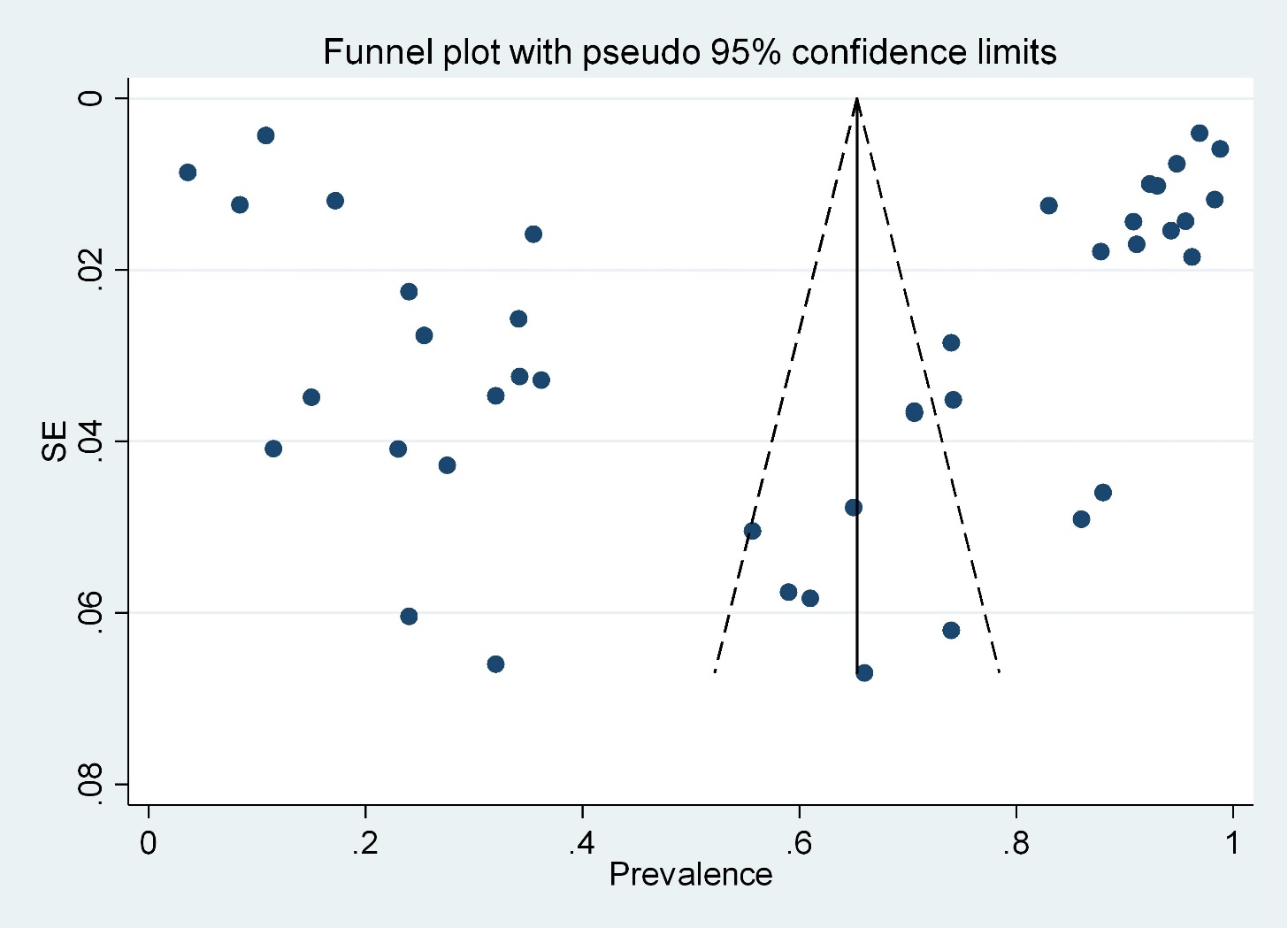


**Figure S2:** Funnel plot for the publication bias of the selected studies.

Each dot of the funnel plot represents a single study. Y-axis represent the standard error of the effect estimates, and the X-axis shows the result (prevalence) for individual study.
